# Supplementary material for: Disruption of O-GlcNAcylation Homeostasis Induced Ovarian Granulosa Cell Injury in Bovine
Source: Int J Mol Sci. 2022 Jul 15;23(14):7815. doi: 10.3390/ijms23147815 (PMC9324263; doi:10.3390/ijms23147815)
Supplement: Supplementary file 1 [file ijms-23-07815-s001.zip › ijms-1756625-supplementary.pdf]

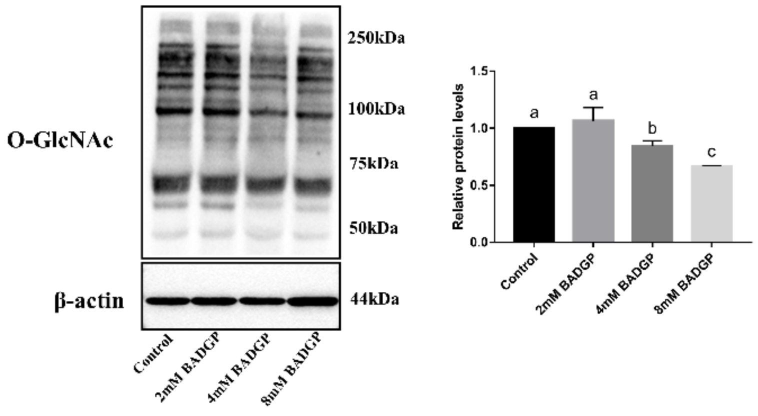

Supplementary Figure S1. Immunoblot of O-GlcNAc levels under different concentrations of BADGP.
